# Bone marrow mesenchymal stem cells derived from juvenile macaques reversed ovarian ageing in elderly macaques

**DOI:** 10.1186/s13287-021-02486-4

**Published:** 2021-08-18

**Authors:** Chuan Tian, Jie He, Yuanyuan An, Zailing Yang, Donghai Yan, Hang Pan, Guanke Lv, Ye Li, Yanying Wang, Yukun Yang, Gaohong Zhu, Zhixu He, Xiangqing Zhu, Xinghua Pan

**Affiliations:** 1 The Basic Medical Laboratory of the 920th Hospital of Joint Logistics Support Force of PLA, The Transfer Medicine Key Laboratory of Cell Therapy Technology of Yunan Province, The Integrated Engineering Laboratory of Cell Biological Medicine of State and Regions, Kunming, 650032 Yunnan Province China; 2 Guizhou Medical University, Tissue Engineering and Stem Cell Experimental Center, Guizhou Provinc Guiyang, 550004 China; 3 Kunming Medical University, Guizhou Province Kunming, 650032 China

**Keywords:** Macaque, BMMSCs, Ovarian ageing

## Abstract

**Background:**

Female sex hormone secretion and reproductive ability decrease with ageing. Bone marrow mesenchymal stem cells (BMMSCs) have been postulated to play a key role in treating ovarian ageing.

**Methods:**

We used macaque ovarian ageing models to observe the structural and functional changes after juvenile BMMSC treatment. Moreover, RNA-seq was used to analyse the ovarian transcriptional expression profile and key pathways through which BMMSCs reverse ovarian ageing.

**Results:**

In the elderly macaque models, the ovaries were atrophied, the regulation ability of sex hormones was reduced, the ovarian structure was destroyed, and only local atretic follicles were observed, in contrast with young rhesus monkeys. Intravenous infusion of BMMSCs in elderly macaques increased ovarian volume, strengthened the regulation ability of sex hormones, reduced the degree of pulmonary fibrosis, inhibited apoptosis, increased density of blood vessels, and promoted follicular regeneration. In addition, the ovarian expression characteristics of ageing-related genes of the elderly treatment group reverted to that of the young control group, 1258 genes that were differentially expressed, among which 415 genes upregulated with age were downregulated, 843 genes downregulated with age were upregulated after BMMSC treatment, and the top 20 differentially expressed genes (DEGs) in the protein-protein interaction (PPI) network were significantly enriched in oocyte meiosis and progesterone-mediated oocyte maturation pathways.

**Conclusion:**

The BMMSCs derived from juvenile macaques can reverse ovarian ageing in elderly macaques.

**Supplementary Information:**

The online version contains supplementary material available at 10.1186/s13287-021-02486-4.

## Introduction

As females age, both fertility and ovarian endocrine function naturally decline due to waning follicle numbers as well as ageing-related cellular dysfunction [1, 2]. Currently, ovarian failure and endocrine disruption are not curable. Societal changes and the increasing desire to preserve fertility have led to various treatment methods, including sex hormone replacement, cytokines, and traditional Chinese medicine (TCM) treatments, to treat ovarian ageing, which regulates fertility and endocrine secretion. However, the long-term use of hormone replacement therapy may cause breast cancer, thrombosis, and other diseases [3]. Cytokine therapy has not yet developed into a large-scale industry and is expensive, characteristics that are not conducive to its widespread application [4]. TCM treatment can partially improve ovarian function, but TCM drug compositions have not been fully elucidated, and there are many uncertain factors [5]. Although assisted reproductive technologies (ARTs) and the “freeze-all” strategy of cryopreserving all oocytes or good-quality embryos have increased the range of options [6], the overall success rate for older women remains very low. Therefore, it is necessary to seek new and effective treatment methods.

Ageing ovaries manifest mainly with tissue atrophy, functional degeneration, insufficient self-renewal ability of reproductive helper cells, and decreased secretion of sex hormones. Bone marrow mesenchymal stem cells (BMMSCs) have multidirectional differentiation potential, a strong self-renewal capacity, and biological characteristics of exosomes secreted with various cytokines [7], and they may become a new tool to delay or reverse ovarian ageing [8]. Many clinical and basic studies have shown the effectiveness of mesenchymal stem cells (MSCs) in the treatment of ovarian ageing, and MSCs have been demonstrated to be more effective than other cell types in improving ovarian function [9]. Human amniotic fluid MSCs (hAFMSCs) can restore ovarian physiological ageing (OPA) function [10]. Human placental MSCs (hPMSCs) can inhibit oxidative stress and apoptosis, thereby improving ovarian function [11]. Exosomes secreted by human umbilical cord MSCs (hUC-MSCs) have a stimulatory effect on primordial follicles and accelerate follicular development [12]. These findings show that MSCs can regulate the secretion of female sex hormones and improve ovarian structure.

However, to date, research on animal models for BMMSC-mediated treatment of ageing and other diseases has focused on small- and medium-sized animals, and there are few studies on primates; furthermore, systematic and standardized studies are lacking. Therefore, in this study, we used a macaque ovarian ageing model as a research object and observed the structural and functional effects of juvenile macaque BMMSCs on ageing macaque ovaries. In addition, we explored the molecular regulatory mechanism by which BMMSCs reverse macaque ovarian ageing. This work provides a theoretical basis and a reference technical solution for the use of BMMSCs to treat ovarian ageing

## Materials and methods

### Materials

#### Macaques and BMMSC sources

Macaques were provided by the Kunming Institute of Zoology, Chinese Academy of Sciences, and the experiments were performed at the Cell Biological Therapy Center of the 920th Hospital of the Chinese People’s Liberation Army. The BMMSCs of juvenile male macaques were provided by our laboratory.

### Methods

#### Evaluation of ovarian ageing models in the elderly macaques

Ovarian ageing models were evaluated according to the age, back and facial features, level of sex hormones, and ovarian morphological structure. Female macaques aged between 22 and 26 years old were used as the elderly group, while young female macaques aged between 6 and 8 years old were used as the control group. Five millilitres of whole blood was intravenously drawn and centrifuged to obtain serum, and 0.5 mL of supernatant was aspirated into a Unicel DXI800 Access Immunoassay System to detect the levels of sex hormones. Anesthetized macaques were used to remove ovarian tissues that were divided for size and morphological analysis and haematoxylin-eosin (HE) staining. Finally, ten healthy elderly female macaques and 5 healthy young female macaques were screened (see supplementary 2 for detailed steps).

#### Preparation of BMMSCs

BMMSCs of 2- to 3-year-old macaques were isolated and cultured by the adherence method. The morphology and growth characteristics of P0 to P4 BMMSCs were observed. P4 BMMSCs were used for flow cytometric analysis to determine the proportion of BMMSC surface antigens and for adipogenic, osteogenic, and chondrogenic induction and differentiation experiments based on methods published previously by our research group [13–17].

#### Macaques grouping and BMMSC transplantation treatment

According to the advice of breeding experts from the Kunming Institute of Zoology, Chinese Academy of Sciences, 10 elderly macaques were randomly divided into an elderly model group (*n* = 4) and an elderly treatment group (*n* = 6), and the remaining 5 macaques formed the young control group (*n* = 5). The P4 BMMSCs were diluted with 0.9% sterile sodium chloride solution to a concentration of 2 × 10^6^ cells/mL. After the macaques of the treatment group had been fixed, BMMSCs were infused into via a femoral vein at a dose of 1 × 10^7^ cells/kg per macaque once every other day for a total of 3 infusions. The macaques in the control and model groups were administered equal volumes of 0.9% sterile sodium (see supplementary 2 for detailed steps).

#### PET-CT observation of ovarian structure and function

Before the experiment, the macaques were fasted for 6 h, injected intravenously with ^18^F-FDG at a dosage of 3.70–4.44 MBq/kg for 60 min, and subjected to whole-body scanning with a GE Discovery^TM^ PET/CT Elite system. CT was conducted using conventional whole-body spiral scanning with the following conditions: tube voltage 120 kV, tube current 240 mA, pitch 0.561, rotation speed 0.5 s/week, layer thickness 3.75 mm, and spacing 512 × 512. PET scanning was conducted with one bed position for 2 min. BestDicom software was used to analyse the different cross-sections, and the maximum standardized uptake value (SUVmax) and CT value were recorded.

#### Detection of sex hormone levels in peripheral blood

Five millilitres of peripheral blood was collected into a heparin tube at 3, 6, and 8 months after BMMSC treatment and centrifuged at 1500 r/min for 5 min. The supernatant was transferred to a 1.5-mL EP tube and centrifuged at 3000 r/min for 3 min; 0.5 mL of the supernatant was then added to the Unicel DXI800 Access Immunoassay System to detect the expression levels of AMH, hFSH, hLH, PRL, Prog, Testo, and E_2_.

#### Collection of macaque ovarian tissues

At 8 months after BMMSC treatment, the macaques were euthanized by anaesthesia with 3% sodium pentobarbital. The abdominal cavity was exposed, to find the uterus, then along the fallopian tube to find the position of the ovary and take it out, weighed (g) ovary on an electronic balance, and imaged. One ovary was sectioned in the horizontal and vertical directions into 4 pieces approximately 1 mm^3^ in size. Two of the sections were placed in a cryopreservation tube, to which 1.8 mL RNA protection solution was added, then stored in liquid nitrogen for transcriptome sequencing. The remaining two sections were fixed in 4% paraformaldehyde solution, dehydrated, embedded in paraffin, and sectioned at a thickness of approximately 4 μm for subsequent histopathological tests.

#### Determination of the histological structure of macaque ovarian tissues after BMMSC treatment

HE staining was performed to observe ovarian structure and follicles, Masson staining was performed to observe the degree of fibrosis, a TUNEL assay was performed to analyse apoptosis, immunohistochemical staining was used to observe the blood vessels, and immunofluorescence staining was performed to track BMMSCs (see supplementary file 1).

#### Transcriptome sequencing of ovarian tissue

Ovarian tissue was ground and lysed, and total RNA was extracted and sequenced. Raw data were obtained by high-throughput sequencing, and the reads were processed by adapter removal and quality control to obtain clean reads. FastQC was used to analyse the quality of sequencing data and obtain relevant information. Htseq-count was used to count the number of reads of some units in the genome. Differential expression analysis was performed with DESeq2. The GO and KEGG annotations of the identified differentially expressed genes (DEGs) were analysed, and Fisher’s exact test was used to calculate the significance level of each GO and pathway term.

#### Statistical analysis

Statistical analyses were performed using SPSS 21.0. The data are expressed as the mean ± standard deviation. The statistical significance between elderly model and young control group, and elderly treatment and model group was performed by *T* test.

## Results

### Evaluation of the macaque research models of ovarian ageing

**Fig. 1:**
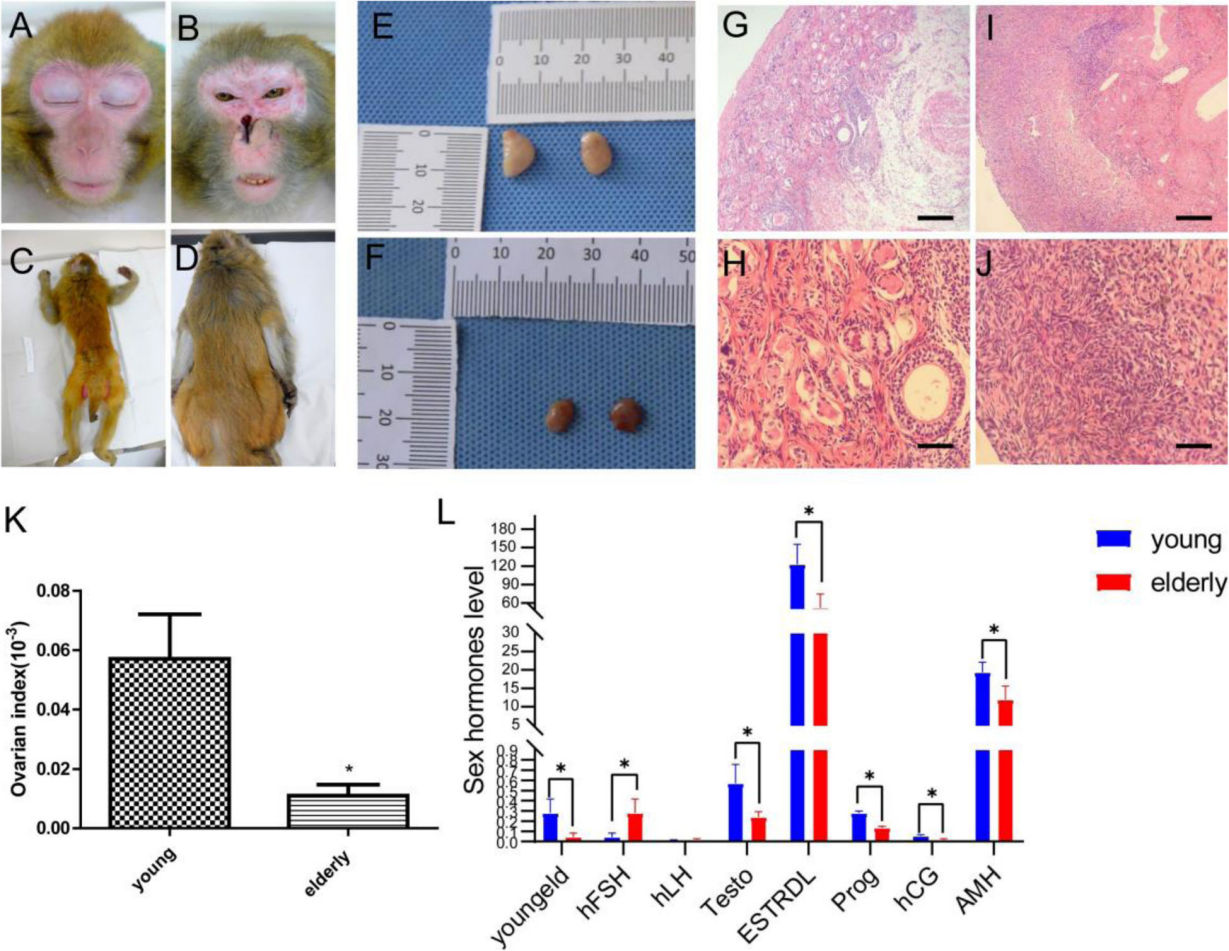
Evaluation of elderly macaques as research models of ovarian ageing. **a** Facial features of young macaques. **b** Facial features of elderly macaques. **c** Back features of young macaques. **d** Back features of elderly macaques. **e** Ovarian morphology of young macaques. **f** Ovarian morphology of elderly macaques. **g** HE staining of a young macaque ovary (100×). **h** HE staining of a young macaque ovary (400×). **i** HE staining of an elderly macaque ovary (100×). **j** HE staining of an elderly macaque ovary (400×). **k** Statistical analysis of ovarian organ index of young and elderly macaques. **l** Statistical analysis of sex hormone secretion levels in young and elderly macaques

The elderly macaques had an average age of 24 years old, weighed 4 to 8 kg, and had a dull coat colour, and their skin was loose and dry, while their faces appeared red (Fig. 1a, c). The sex hormone levels in the elderly macaques were as follows (Fig. 1l): 0.28 ± 0.11 mIU/mL follicle-stimulating hormone (FSH), 0.017 ± 0.009 mIU/mL luteinizing hormone (LH), 0.24 ± 0.042 ng/mL testosterone (Testo), 51.86 ± 18.37 pg/mL oestradiol (E2), 0.13 ± 0.012 ng/mL progesterone (Prog), 0.013 ± 0.012 chorionic gonadotropin (CG), and 11.96 ± 2.96 pmol/l anti-Müllerian hormone (AMH). The ovarian atrophy (Fig. 1f), ovarian organ index (0.011 ± 0.005; Fig. 1k), and HE staining results showed essentially no follicular structure, with only local atretic follicles observed, filled with fat and connective tissue (Fig. 1i, j). The young macaques had an average age of 7 years old, weighed 4 to 8 kg, and had a bright coat colour (Fig. 1b, d). The sex hormone levels in the young macaques were as follows (Fig. 1l): 0.043 ± 0.03 mIU/mL hFSH, 0.007 ± 0.009 mIU/mL hLH, 0.57 ± 0.15 ng/mL Testo, 123.2 ± 26.26 pg/mL E2, 0.28 ± 0.014 ng/mL Prog, 0.05 ± 0.012 hCG, and 11.96 ± 2.96 pmol/l AMH. The ovarian dilatation (Fig. 1e), ovarian organ index (0.057 ± 0.021; Fig. 1k), and the HE staining results showed that all levels of follicles could be observed, with the medulla and interstitial boundaries being easily observed and neatly arranged (Fig. 1g, h).

### Morphology of BMMSCs

**Fig. 2:**
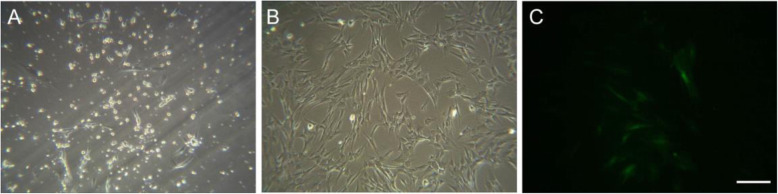
Morphology of BMMSCs. **a** Primary generation of BMMSCs (100×); **b** P4 BMMSCs (100×); **c** E-GFP-labelled BMMSCs (100×)

The growth state of BMMSCs was observed under an inverted fluorescence phase-contrast microscope. The results showed that a small number of primary BMMSCs migrated out in a short spindle shape after 3–4 days, and a large number of suspended impurities were present in the supernatant (Fig. 2a). The P4 fibroblast-like BMMSCs were densely arranged in a spiral pattern and exhibited a long spindle shape, obvious directionality, typical cell morphology characteristics, uniform morphology, and a strong refractive index (Fig. 2b). Subsequently, the P4 BMMSCs were labelled with and expressed enhanced green fluorescent protein (E-GFP) (Fig. 2c).

### Changes in ovarian tissue structure and sex hormones after BMMSC treatment

**Fig. 3:**
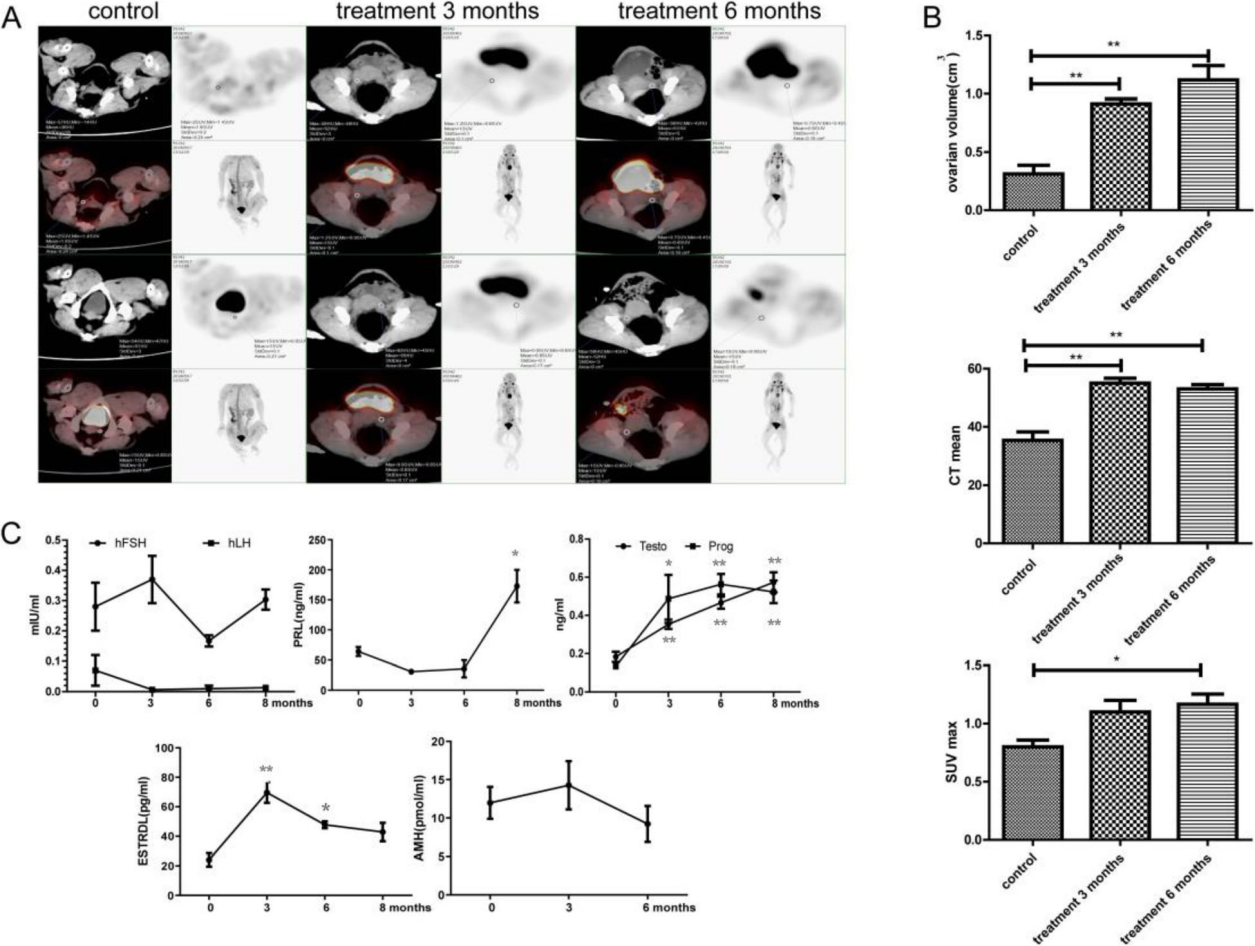
Changes in ovarian structure and function after BMMSC treatment. **a** PECT-CT was used to observe the changes in ovarian SUVmax and volume and the CT value; white areas represent the ovarian volume, and red areas represent metabolically active regions. **b** Statistical analysis of CT value, volume, and SUVmax. **c** Changes in hFSH, hLH, Testo, Prog, PRL, E2, and AMH levels at 3, 6, and 8 months after BMMSC treatment

PET-CT was used to analyse changes in ovarian volume, SUVmax, and CT value (Fig. 3a). Ovarian volume was 0.31 ± 0.11 cm^3^ at 0 months (control), 1.43 ± 0.73 cm^3^ at 3 months, and 1.12 ± 0.18 cm^3^ at 6 months (Fig. 3b). SUVmax was 0.8 ± 0.08 at 0 months (control), 1.4 ± 0.43 at 3 months, and 1.17 ± 0.12 at 6 months (Fig. 3b). CT value was 35.33 ± 4.11 at 0 months (control), 55 ± 2.45 at 3 months, and 53 ± 2.16 at 6 months (Fig. 3b).

The major functions of the ovaries are to govern the health of the female by regulating endocrine status and the production of mature oocytes [18]. Therefore, the sex hormone levels in peripheral blood were assessed to evaluate the effects of BMMSCs on ageing ovaries. Compared to those before treatment, the levels of Prog and Testo were significantly increased at 3, 6, and 8 months (Fig. 3c; *p* < 0.01). The PRL level was significantly increased relative to the 0-month level at 8 months (Fig. 3c; *p* < 0.05). The E2 level was significantly increased (*p* < 0.01) at 3 months and had decreased by 6 and 8 months but remained higher than the level at 0 months (Fig. 3c; *p* < 0.05). However, the levels of hFSH, hLH, and AMH were not significantly different (Fig. 3c; *p* > 0.05) before and after BMMSC treatment.

### Ovarian histopathological structure was improved and follicular regeneration occurred after BMMSC treatment

**Fig. 4:**
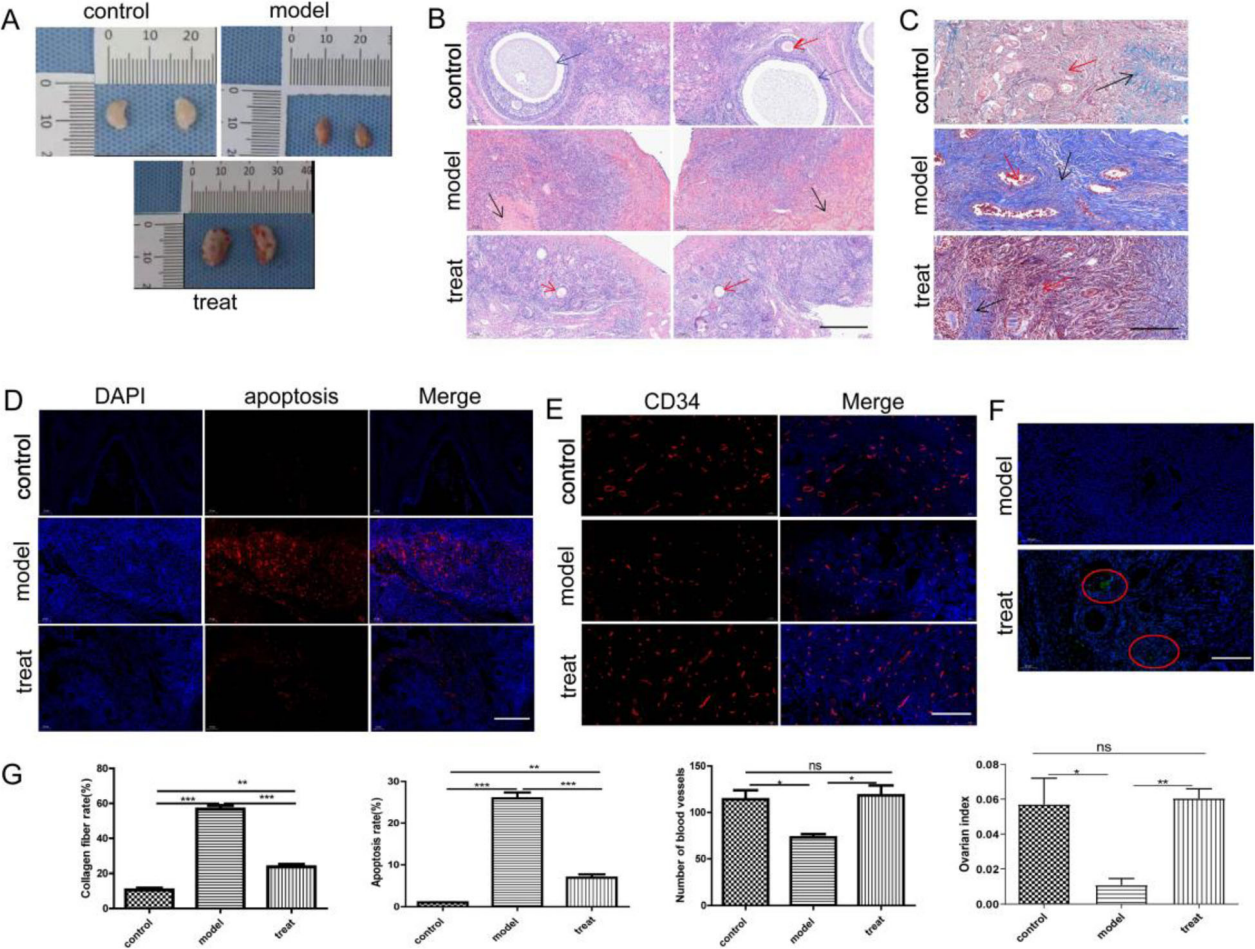
Ovarian histopathological observation after BMMSC treatment. **a** The morphology of ovaries. **b** HE staining was performed to observe ovarian structure (100 μm). **c** Masson staining was performed to observe the degree of fibrosis (50 μm). **d** TUNEL assay was performed to analyse apoptosis (100 μm). **e** Immunohistochemical staining was performed to observe the density of blood vessels (50 μm). **f** Immunofluorescence staining was performed to track BMMSCs in the ovary (50 μm). **g** Statistical analyses of the degree of fibrosis, the percentage of apoptotic cells, the density of blood vessels, and the ovarian organ index (^*^p < 0.05, ^**^p < 0.01, and ^***^p < 0.001)

Folliculogenesis is a precise and orderly process of internal coordination and external regulation in women [18]. A decline in ovarian function characterized by a decrease in both the quantity and quality of primordial follicles occurs with ageing [12]. In the present study, the changes in ovarian histopathological structure reflected the therapeutic effect of BMMSCs. Interestingly, ovary morphology was improved after BMMSC treatment (Fig. 4a). HE staining was used to visualize ovarian structures (Fig. 4b). In the young control group, primordial, primary, secondary (red arrows), and mature follicles (blue arrows) were observed, and contextual interstitial communication was obvious. In the elderly model group, no obvious follicle structure was observed, and large amounts of connective tissue (green arrows) and brown-yellow pigment deposition (black arrows) were observed in local areas. In the elderly treatment group, a number of primordial, primary, secondary (red arrows), and atretic follicles were observed, with clear ovarian structure.

Fibrosis is a hallmark of ageing tissues, and the ovary is the first organ to show overt signs of ageing. Recent studies have demonstrated that ageing often leads to altered ovarian architecture and function, including increased fibrosis in the ovarian stroma. MSC transplantation has been shown to be an effective method to inhibit ovarian fibrosis and restore ovarian function [19, 20]. Masson staining was performed to observe the degree of fibrosis (Fig. 4c); in the figure, blue represents collagen fibres, and red represents cellulose. The percentage of collagen fibres was 10.61 ± 1.83% in the young control group. 56.79 ± 3.58% in the elderly model group, the deposition area was large, well-arranged, and disordered, and with few muscle fibres located locally. 23.71 ± 2.4% in the elderly treatment group, the fibres were mostly deposited in the cortex layer, the deposition area was small, and the arrangement was loose (Fig. 4c, e).

Follicular atresia is related to the apoptosis of granulosa cells, which are large in ovarian follicles; previous studies showed that MSCs improved the apoptosis [21, 22]. TUNEL assay was performed to analyse apoptosis, with red staining indicating apoptotic cells (Fig. 4d). The apoptosis rate was 1.07 ± 0.04% in the young control group, 25.93 ± 2.49% in the elderly model group, and 6.98 ± 1.35% in the elderly treatment group (Fig. 4g).

Previous studies have revealed that MSCs augment the density of FITC-dextran perfused blood vessels [23] and that intravenous injection of preconditioned MSCs improves microvascular dynamics [24]. We performed immunohistochemical staining to observe the density of blood vessels, with CD34-positive granules indicating blood vessels (Fig. 4e). The density of blood vessels was 114 ± 17 in the young control group, 73 ± 6 in the elderly model group, and 118 ± 18 in the elderly treatment group (Fig. 4g).

From the changes of ovarian histology after BMMSC treatment can be seen that BMMSCs improve ovarian structure and function, in order to assess whether BMMSCs exhibit homing to ovaries, immunofluorescence staining was performed to track BMMSCs in the ovary. Two immunofluorescent granules were detected in the elderly treatment group, while no immunofluorescence was observed in the elderly model group (Fig. 4f).

A total of 1258 genes were differentially expressed, and ageing-related genes partly returned to a young phenotype following BMMSC treatment, with the function correlated to Prog-mediated oocyte maturation.

**Fig. 5:**
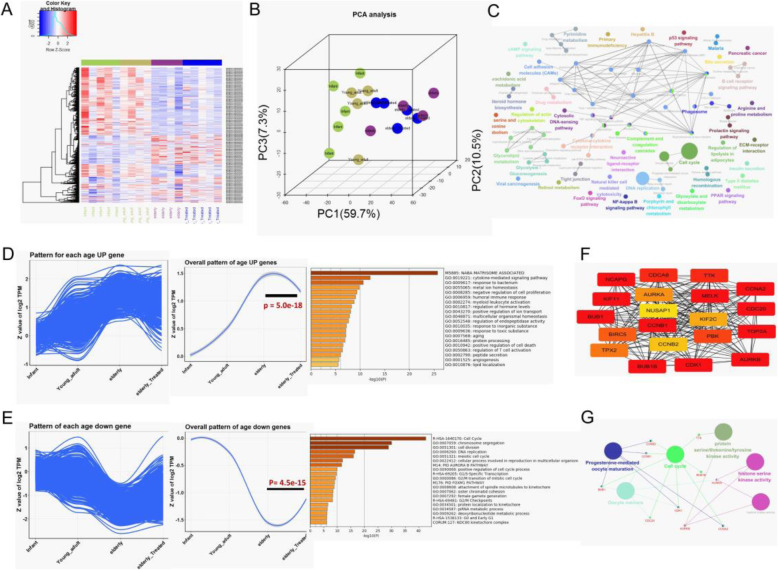
Transcriptome sequencing of ovarian tissues. **a** Cluster plot showing the 1258 genes differentially expression. **b** 3D-PCA analysis of ageing-related genes, light green represents the young control group, purple represents the elderly model group, and dark blue represents the elderly treatment group. **c** ClueGO analysis functional enrichment of 1258 genes. **d** Trajectory analysis the ovarian expression characteristics of 415 upregulated genes with age and BMMSC treatment, and the GO enrichment. **e** Trajectory analysis of the ovarian expression characteristics of 843 downregulated genes with age and BMMSC treatment, and the GO enrichment. **f** Cytohub screened the top20 PPI. **g** PPI top 20 gene function and pathway

After observing the effects of BMMSCs on ovarian ageing with respect to ovarian tissue structure and the secretion of sex hormones, RNA-seq was performed on ovarian tissue to identify key genes and signalling pathways. Cluster plots showed that 1258 genes were differentially expressed after BMMSC treatment (Fig. 5a). 3D-PCA trajectory analysis showed that the ovarian expression characteristics of ageing-related genes of the elderly treatment group reverted to that of the young control group (Fig. 5b). GO analysis showed that the DEGs were primarily enriched in terms related to the cell cycle (Fig. 5c). A total of 415 genes were upregulated with ageing and downregulated after BMMSC treatment (Fig. 5d) (*p* = 5.0e−18). A total of 843 genes were downregulated with ageing and upregulated after BMMSC treatment and were enriched in the NABA matrisome-associated and cytokine-mediated signalling pathways and metal ion homeostasis (Fig. 5e) (*p* = 4.5e−154). CytoHubba analysis revealed the top 20 DEGs in the protein-protein interaction (PPI) network (Fig. 5f), and ClueGO analysis showed that these DEGs were enriched primarily in the terms of cell cycle, oocyte meiosis, progesterone-mediated oocyte maturation, histone serine kinase activity, and protein threonine/histone/tyrosine serine kinase pathway (Fig. 5g).

## Discussion

Ovarian ageing weakens female reproduction, ovulation, secretion of sex hormones, and other functions and affects the tissues and organs of the body. It is a gradual, multi-factorial, and complex biological process caused by the combined effects of the decreasing number and quality of follicles. MSC transplantation has been shown to be effective and safe as a new therapeutic method for ovarian ageing [25], which proposed to restore ovarian structure and function [26]. Interestingly, our results provide a comprehensive understanding of the regulation of BMMSC interaction with ovarian ageing.

In our study, PET-CT showed that ovarian volume increased, lesions decreased, and metabolism was vigorous after BMMSC treatment. Sex hormones are secreted by ovaries to carry out specific functions and affect other organs. Our results showed that Prog, Testo, PRL, and E2 were significantly increased after BMMSC treatment, while FSH, LH, and AMH were not significantly different before and after BMMSC treatment. These results are consistent with previous studies reporting the ability of MSCs to restore ovarian structure and sex hormone secretion [22, 27, 28]. Furthermore, previous studies have demonstrated that the number of MSCs in different cell cycle stages can be adjusted by adjusting the concentrations of sex hormones [29, 30], suggesting that after BMMSCs restore the secretion of sex hormones, sex hormones may in turn regulate the biological function of MSCs.

In our study, a comparative analysis of the HE staining results between the elderly treatment and model groups showed that BMMSCs improved ovarian structure and promoted follicle regeneration. These results are consistent with these reported in previous studies on MSC treatment of ovarian structure destruction and functional decline [28, 31, 32]. Interestingly, a previous study demonstrated the presence of adult oogonial stem cells (OSCs) in the adult axolotl salamander ovary and showed that ovarian injury induces OSC activation and functional regeneration of the ovaries [33]. In addition, OSC activation resulted in rapid differentiation into new oocytes, and follicle cell proliferation promoted follicle maturation during ovarian regeneration [34]. These results indicate that transplanted BMMSCs home to the ovaries or function via the paracrine pathway to regulate the ovarian microenvironment to activate OSCs, thereby promoting follicle regeneration and improving ovarian structure.

Ovaries typically become fibrotic with ageing, which leads to ovarian structural dysfunction and function decline [35]. Therefore, alleviating or reversing fibrotic ovaries is a strategy to treat ovarian ageing. In our study, Masson staining showed that the degree of fibrosis was significantly decreased after BMMSC treatment. Previous studies have shown similar effectiveness of MSCs in inhibiting ovarian fibrosis [20, 36], and the mechanism involves mainly MSC-mediated inhibition of inflammatory factors [37]. These results suggested that BMMSCs inhibit the inflammatory response by secreting various immune and inflammation regulatory factors to reduce the degree of ovarian fibrosis. However, their regulation of ovarian tissue fibrosis has not been shown to restore to the level observed in young macaques.

In this study, our TUNEL assay showed that apoptosis was significantly decreased after BMMSC treatment. These results are consistent with these reported in previous studies on MSCs inhibited apoptosis to treat ageing-related diseases [38–40]. Additionally, a study of MSC-treated follicle loss has shown that MSCs suppressed the expression of apoptotic genes and had antiapoptotic effects [41]. These results suggest that BMMSCs reduced the apoptosis of ageing ovarian cells to balance cell proliferation with apoptosis, to increase the number of reproductive helper cells.

Our RNA-seq analysis of ovarian tissue identified 1258 genes that were differentially expressed, 415 of which were genes upregulated with age and downregulated after BMMSC treatment, and 843 of which were genes downregulated with age and upregulated after BMMSC treatment, and the ovarian expression characteristics of ageing-related genes partly returned to a young phenotype following BMMSC treatment. Moreover, the top 20 DEGs in the PPI were primarily enriched in the terms of cell cycle, oocyte meiosis, and progesterone-mediated oocyte maturation; these results suggest that the ovarian transcriptional expression profile of rhesus monkeys in the elderly treatment group shifted to a younger direction, and the BMMSCs derived from juvenile macaques could fully reverse the process of ovarian ageing at the molecular level and significantly reduce the content of ageing-related molecules. Interestingly, the top 20 DEGs in the PPI are detrimental to maintaining ovarian structure and function, in particular, the enrichment in the pathways involving oocyte meiosis and Prog-mediated oocyte maturation were consistent with the findings of our in vivo experiments, which demonstrated that ovarian structure was improved, new follicles appeared, and Prog levels increased steadily after BMMSC treatment, which indicate that the Prog-mediated oocyte maturation pathway plays a key role in the reversal of ovarian ageing by BMMSCs, and that the associated genes CCNB1, CCNB2, CCNB1, BUB1, CDC20, and CDK1 may become new therapeutic targets in BMMSC treatment of ovarian ageing.

In summary, BMMSCs regulate the secretion of sex hormones, suppress cell apoptosis, inhibit the degree of fibrosis, reverse the process of ovarian ageing at the molecular level, and significantly reduce the content of ageing-related molecules; these effects restore ovarian structure and function, to promote follicle and blood vessel regeneration.

## Conclusions

i. In the elderly macaque model of ovarian ageing, the ovarian organ index was decreased; ovarian atrophy and structural destruction occurred, with only local atretic follicles observed; FSH and LH levels were increased, while Testo, E2, Prog, CG, and AMH levels were decreased.

ii. BMMSCs derived from juvenile macaques reversed ovarian ageing in elderly macaques, by promoting follicle and blood vessel regeneration, restoring ovarian structure, suppressing cell apoptosis, inhibiting the degree of fibrosis, and restoring the normal secretion of sex hormones.

iii. BMMSCs derived from juvenile macaques reversed the process of ovarian ageing at the molecular level and significantly reduce the content of ageing-related molecules; the oocyte meiosis and Prog-mediated oocyte maturation pathways play key roles in BMMSC treatment of ovarian ageing.

## Supplementary Information

**Additional file 1:.** HE staining of ovarian tissue, Masson staining of ovarian tissue, immunofluorescence staining of ovarian tissue, TUNEL staining of ovarian tissue, and immunohistochemical staining to detect CD34 in ovarian tissue

**Additional file 2:.** Evaluation of ovarian ageing models in elderly macaques and macaque grouping and BMMSC transplantation treatment

## Data Availability

All data generated or analysed during this study are included in this published article.

## Abbreviations

BMMSCs: Bone marrow mesenchymal stem cells
hFSH: Human follicle-stimulating hormone
Prog: Progesterone
Testo: Testosterone
E2: Oestradiol
^18^F-FDG: β-2-[18 F]-Fluoro-2-deoxy-d-glucose
FBS: Foetal bovine serum
