# Supplementary material for: Bone marrow mesenchymal stem cells derived from juvenile macaques reversed ovarian ageing in elderly macaques
Source: Stem Cell Res Ther. 2021 Aug 18;12:460. doi: 10.1186/s13287-021-02486-4 (PMC8371769; doi:10.1186/s13287-021-02486-4)
Supplement: Supplementary file 1 — Additional file 1:. HE staining of ovarian tissue, Masson staining of ovarian tissue, immunofluorescence staining of ovarian tissue, TUNEL staining of ovarian tissue, and immunohistochemical staining to detect CD34 in ovarian tissue [file 13287_2021_2486_MOESM1_ESM.docx]

**HE staining of ovarian tissue**

The sections were put into xylene I for 20 min, xylene II for 20 min, absolute ethanol I for 5 min, absolute ethanol II for 5 min, and 75% alcohol for 5 min and washed 3 times with tap water. Then, put it into 5 mL of haematoxylin staining solution, dye for 3~5 minutes, wash 3 times with tap water, differentiate the differentiation solution for 5 minutes, wash 3 times with tap water, return the blue solution to blue for 5 minutes, and rinse slowly with running water 3 times. Then, the slices were placed into 85% and 95% gradient alcohols, dehydrated for 5 minutes each, and added to the eosin staining solution for 5 minutes. Then, the sections were put into absolute ethanol I for 5 min, absolute ethanol II for 5 min, absolute ethanol III for 5 min, xylene I for 5 min, and xylene II for 5 min for transparency, and the slides were sealed with neutral gum. Finally, microscopic examination, image acquisition and analysis were performed.

**Masson staining of ovarian tissue**

First, the sections were placed in xylene 2 times for 20 min, absolute ethanol 2 times for 5 min, and 75% alcohol for 5 min. Then, the slices were immersed in Masson A solution, soaked overnight, and washed with tap water 3 times the next day. After the slices were immersed for 1 min, they were washed with tap water 3 times, differentiated with 1% hydrochloric acid and alcohol, and washed with tap water 3 times. The slices were placed in Masson D solution for 6 min, rinsed with tap water 3 times, and then Masson E solution was used for 1 min. After slight draining, the slices were placed directly in Masson F solution and dyed for 20-30 s. Then, the sections were rinsed and differentiated with 1% glacial acetic acid, and two cylinders of absolute ethanol were dehydrated. Then, the slices were placed in the third container of absolute ethanol for 5 min, cleared with xylene for 5 min, and sealed with neutral gum. Finally, microscopic inspection, image acquisition and analysis were performed.

**TUNEL staining of ovarian tissue**

The frozen section was placed horizontally at 37°C for 15 min, 4% paraformaldehyde for 30 min, and PBS (pH 7.4) for 15 min. The sections were decolorized by shaking and washing on a decolorizing shaker 3 times for 5 min each. The proteinase K working solution was dropped in the circle to cover the tissue, and the section was incubated for 25 min in a 37°C incubator. Then, the slides were placed in PBS and washed with shaking on a decolorizing shaker 3 times for 5 min each time. The membrane rupture working solution was added to cover the tissues, and the samples were incubated at room temperature for 20 min. Furthermore, the slices were placed in PBS, shaken and washed 3 times for 5 min each time. Then, 1x Equilibration Buffer was added dropwise to cover the entire sample area to be tested, and the samples were incubated at room temperature for 10 min. The reaction solution was added: Recombinant TDT Enzyme, BrightRed Labelling Mix, 5x Equilibration Buffer and deionized water at a 1:5:10:34 ratio, and the solution was slowly dropped into the circle to cover the tissue. Subsequently, the slices were laid flat in a wet box and incubated for 2 h in a 37°C incubator. After fully aspirating the PBS, DAPI staining solution was added dropwise to the circle and incubated at room temperature in the dark for 10 min. The slices were placed in PBS, washed and shaken 3 times for 5 min each time. After the slices were slightly dried, the tablets were mounted with anti-fluorescence quenching. Finally, Microscopic examination.

**Immunohistochemical staining to detect CD34 in ovarian tissue**

The sections were put into xylene 2 times for 20 min, absolute ethanol 2 times for 5 min, 75% alcohol for 5 min, and washed with double distilled water 3 times. The tissue sections were placed in EDTA antigen retrieval solution, placed in a microwave oven for 8 min, stopped for 8 min, turned to medium and low power for 7 min for antigen retrieval. The slides were washed in PBS 3 times for 5 min each time. BSA was added dropwise, and the samples were incubated for 30 min. CD34 antibody (1:3000) was added, incubated overnight and then washed 3 times in PBS for 5 min each time. The secondary antibody was added and incubated for 50 min at room temperature in the dark. An anti-fluorescence quenching mounter was added to the centre of the circle to mount the slide. DAPI dye solution was added dropwise to the circle and incubated for 10 min at room temperature in the dark. The sections were washed 3 times with PBS for 5 min each time. According to the proofreading counting method of Weidner *et al*, blood vessels were counted.

**Immunofluorescence staining of ovarian tissue**

The sections were placed in xylene 2 times for 15 min, absolute ethanol 2 times for 5 min, 85% alcohol for 5 min, 75% alcohol for 5 min, and then washed three times with distilled water. Then, draw a circle with a tissue brush around the position of the tissue section. DAPI staining solution was added dropwise to the circle and incubated at room temperature for 10 min in the dark. The slices were placed in PBS, shaken and washed 3 times for 5 min each time on a decolorizing shaker. After the slices were slightly dried, anti-fluorescence quenching and blocking tablets were added for mounting. Finally, the slices were observed under a fluorescence microscope and then scanned.
