# Supplementary material for: Bone marrow mesenchymal stem cells derived from juvenile macaques reversed ovarian ageing in elderly macaques
Source: Stem Cell Res Ther. 2021 Aug 18;12:460. doi: 10.1186/s13287-021-02486-4 (PMC8371769; doi:10.1186/s13287-021-02486-4)
Supplement: Supplementary file 2 — Additional file 2:. Evaluation of ovarian ageing models in elderly macaques and macaque grouping and BMMSC transplantation treatment [file 13287_2021_2486_MOESM2_ESM.doc]

Evaluation of ovarian ageing models in elderly macaques

(1) According to the age, sex hormone secretion level and ovarian morphology of rhesus monkeys, 10 healthy, old female rhesus monkeys, with an average age of 24 years old, and 5 healthy, young female rhesus monkeys weighing 4-8 kg, with an average age of 7 years and a body weight of 4.5-8 kg, were selected.

(2) Young and old rhesus monkeys were anaesthetized with 3% pentobarbital sodium (1 kg/mL) and fixed on an operating table for 15 min. The blood collection sites of the rhesus monkeys were disinfected with 75% alcohol. Five millilitres of whole blood was collected from the femoral vein by the vacuum blood collection method, and 3 mL of whole blood was collected in a heparin tube and stored for 2 h. After whole blood coagulation and centrifugation at 1000 rpm for 5 min, the supernatant was separately packed and transferred to a 1.5-mL EP tube, and the supernatant was sucked into a Unicel DXI800 Access Immunoassay System and centrifuged at 3000 rpm for 3 min. The expression levels of AMH, FSH, LH, hCG, progesterone, testosterone and E2 were detected.

(3) The rhesus monkeys were anaesthetized with excess 3% pentobarbital sodium to euthanize them; then, the following steps were performed: first open the abdominal cavity, find the uterus, then along the fallopian tube to find the position of the ovary and peel it off. Second cut a circle around the vagina, and finally remove the uterus, fallopian tubes and ovaries from the outside of the vagina. Ovary was weigh (g) and took pictures on the electronic balance, and assess the ovarian organ index of the old and young rhesus monkeys. After the ovaries were separated, one ovary was cut into 2 parts horizontally and vertically, approximately 1 mm^3^ in size, and fixed in 4% paraformaldehyde solution for 24 h. The ovarian tissue was dehydrated, embedded in paraffin and sliced at a thickness of approximately 4 μm. The ovarian tissue structure was observed by HE staining.

(4) HE staining of ovarian tissue: the sections were washed for 20 min in xylene Ⅰ, 20 min in xylene Ⅱ, 5 min in anhydrous ethanol Ⅰ, 5 min in anhydrous ethanol Ⅱ and 75% alcohol, and 5 min in tap water 3 times. Then, the sections were put into 5 mL of haematoxylin dye solution and washed with tap water 3 times for 5 min each, rinsed with tap water 3 times, and rinsed slowly with running water 3 times for 5 min. The slices were then put into 85% and 95% gradient alcohol solutions, dehydrated for 5 min, and stained for 5 min in eosin dye solution. Then, the slices were put into anhydrous ethanol I for 5 min, anhydrous ethanol II for 5 min, anhydrous ethanol III for 5 min, xylene I for 5 min, xylene II for 5 min until the sections were transparent, and sealed with neutral gum. Finally, microscope examination, image acquisition and analysis were performed.

Macaque grouping and BMMSC transplantation treatment

(1) Ten old and 5 young rhesus monkeys were fed normally for one week, and no abnormal changes were observed. According to the advice of feeding experts of Kunming Institute of Zoology, Chinese Academy of Sciences, 10 old rhesus monkeys were randomly divided into three groups: aged model group (n = 4), aged treatment group (n = 6) and young control group (n = 5).

(2) P3 generation bone marrow mesenchymal stem cells were extracted from liquid nitrogen and thawed rapidly at 37 ℃. A cryopreserved tube was inoculated in a T175 culture bottle at 1:3 dilution and cultured in a cell incubator with 5% CO_2_, 37 ℃ and 100% humidity for 24 hours. When the fusion degree of the cells reached 75%, the supernatant was poured out, and the cells were washed twice with 10 mL of aseptic saline and digested for 3 min at 37 ℃ with 3 mL of 0.25% EDTA trypsin. Then, 6 mL of DMEM/F12 medium containing 10% foetal bovine serum was added to stop digestion, fully mixed, and centrifuged at 1000 rpm for 3 min. The supernatant was then discarded, 6 mL of DMEM/F12 medium containing 10% foetal bovine serum was added, the cells were re-suspended and mixed, and they were inoculated in T175 cell culture flasks and cultured at 5% CO2, 37 ℃, and 100% humidity in a cell culture box.

（3）When the fusion degree of P4 generation bone marrow mesenchymal stem cells in T175 cm2 flasks was approximately 90%, the supernatant was discarded, cells were washed with 15 mL of saline 3 times, the saline was discarded, and 3 mL of 0.25% EDTA trypsin was added for 3 min. Then, 6 mL of basic medium was added to terminate digestion, the suspension was transferred to a 15-mL centrifuge tube and centrifuged at 2000 r/min for 3 min to absorb the supernatant, 3 mL of saline was added for re-suspension, the suspension was mixed, and 10 μL was taken for cell counting. The cells were diluted with normal saline at a concentration of 2 × 10^6^ cells/mL and finally transferred to a 50-mL centrifuge tube for backup. The above procedure was repeated until a one-time dose of 6 rhesus monkeys in the treatment group was satisfied.

(4) The rhesus monkeys were anaesthetized with 3% pentobarbital sodium. After weighing the rhesus monkeys, they were fixed on the operating table, and the cells for infusion were disinfected with 75% alcohol. According to the cell dose of 107 cells/kg, the volume of bone marrow mesenchymal stem cell suspension needed for each rhesus monkey was calculated according to the body weight of the rhesus monkeys.The cells from the rhesus monkeys in the treatment group were infused into the elderly group through the femoral vein once a day every other day three consecutive times. The same volume of normal saline was infused at the same time in the elderly and young control groups.
